# Considerations for drug trials in hypertrophic cardiomyopathy

**DOI:** 10.1002/ehf2.15138

**Published:** 2024-10-27

**Authors:** John P. Farrant, Matthias Schmitt, Anna B. Reid, Clifford J. Garratt, William G. Newman, Aneil Malhotra, Rhys Beynon, Masliza Mahmod, Betty Raman, Robert M. Cooper, Dana Dawson, Thomas Green, Sanjay K. Prasad, Anvesha Singh, Susanna Dodd, Hugh Watkins, Stefan Neubauer, Christopher A. Miller

**Affiliations:** ^1^ Division of Cardiovascular Sciences, School of Medical Sciences, Faculty of Biology, Medicine and Health, Manchester Academic Health Science Centre University of Manchester Oxford Road Manchester M13 9PL UK; ^2^ Manchester University NHS Foundation Trust Southmoor Road, Wythenshawe Manchester M23 9LT UK; ^3^ Manchester Centre for Genomic Medicine Manchester University NHS Foundation Trust Oxford Road Manchester M13 9WL UK; ^4^ Division of Evolution, Infection and Genomics, School of Biological Sciences, Faculty of Biology, Medicine and Health, Manchester Academic Health Science Centre University of Manchester Oxford Road Manchester M13 9PL UK; ^5^ Institute of Sport Manchester Metropolitan University 99 Oxford Rd Manchester M1 7EL UK; ^6^ Division of Cardiovascular Medicine, Radcliffe Department of Medicine University of Oxford Oxford OX3 9DU UK; ^7^ NIHR Oxford Biomedical Research Centre Oxford University Hospitals Foundation Trust Oxford OX3 9DU UK; ^8^ Liverpool Heart and Chest Hospital Thomas Dr Liverpool L14 3PE UK; ^9^ Liverpool John Moores University 70 Mount Pleasant Merseyside L3 5UX UK; ^10^ School of Medicine University of Aberdeen Aberdeen AB25 2ZD UK; ^11^ Cardiology Department Aberdeen Royal Infirmary Aberdeen AB25 2ZN UK; ^12^ Cardiology Department Northumbria Healthcare NHS Trust Northumberland UK; ^13^ Royal Brompton and Harefield NHS Foundation Trust Sydney St London SW3 6NP UK; ^14^ National Heart and Lung Institute Imperial College London London; ^15^ Department of Cardiovascular Sciences University of Leicester and the NIHR Leicester Biomedical Research Centre, Glenfield Hospital Groby Road Leicester LE3 9QP UK; ^16^ Department of Health Data Sciences, Institute of Population Health, Faculty of Health and Life Sciences University of Liverpool Block F, Waterhouse Boulevard, 1‐5 Brownlow Street Liverpool L69 3GL UK; ^17^ Wellcome Centre for Cell‐Matrix Research, Division of Cell‐Matrix Biology & Regenerative Medicine, School of Biology, Faculty of Biology, Medicine & Health, Manchester Academic Health Science Centre University of Manchester Oxford Road Manchester M13 9PT UK

**Keywords:** Hypertrophic cardiomyopathy, Clinical trials, Patient selection, Trial endpoints, Disease‐modifying therapy

## Abstract

Hypertrophic cardiomyopathy (HCM) is a heterogeneous condition with potentially serious manifestations. Management has traditionally comprised therapies to palliate symptoms and implantable cardioverter‐defibrillators to prevent sudden cardiac death. The need for disease‐modifying therapies has been recognized for decades. More recently, an increasing number of novel and repurposed therapies hypothesized to target HCM disease pathways have been evaluated, culminating in the recent regulatory approval of mavacamten, a novel oral myosin inhibitor. HCM poses several unique challenges for clinical trials, which are important to recognize when designing trials and interpreting findings. This manuscript discusses the key considerations in the context of recent and ongoing randomized trials, including the roles of genotype, phenotype and symptom status in patient selection, the evidence base for clinical and mechanistic outcome measurements, trial duration and sample size.

## Introduction

Hypertrophic cardiomyopathy (HCM) is the most common inherited cardiac disorder, characterized by cardiomyocyte disarray, left ventricular (LV) hypertrophy, small vessel disease and myocardial fibrosis.^1^, ^2^ Clinical manifestations are variable. Two‐thirds of patients have symptoms at diagnosis, around a fifth of patients develop heart failure, and a fifth develop atrial fibrillation, although clinical events vary considerably according to age of diagnosis and presence of a sarcomeric mutation.^3^, ^4^ Approximately 1% of patients experience sudden death or resuscitated cardiac arrest annually.^4^

Management has traditionally comprised therapies to palliate symptoms and implantable cardioverter‐defibrillators (ICD) aiming to prevent sudden cardiac death (SCD). The absence of disease‐modifying therapies led, in 2010, to a Working Group of the National Heart, Lung, and Blood Institute to identify a ‘critical need’ … to … ‘identify putative targets for intervention’ … and to undertake clinical trials … ‘to determine if novel (or currently available) drug therapies can target these pathways of HCM disease expression and, thereby, improve on the natural history of patients’.^5^

Since then, an increasing number of novel and repurposed therapies hypothesized to target HCM disease pathways have been evaluated in randomized controlled trials (*Table* *1*), culminating in the recent regulatory approval of mavacamten, a novel oral myosin inhibitor, for adults with symptomatic New York Heart Association (NYHA) class II–III obstructive HCM to improve exercise capacity and symptoms.^27^, ^28^ There nevertheless remains a substantial unmet need for patients with HCM in terms minimizing phenotype progression, improving quality of life and reducing the risk of adverse clinical events.

**Table 1:** Design, putative disease pathway being targeted and key enrolment criteria of completed and ongoing placebo‐controlled randomized trials in HCM

Trial/authors (Year of publication)	Design	Treatment duration	Putative/hypothesized disease pathway targeted	Key inclusion criteria
CHANCE^6^ (2009)	1:1 candesartan (32 mg daily): placebo	12 months at maintenance dose	Inhibition of angiotensin II type 1 receptor	Age ≥18 years LV wall thickness >15 mm LVEF ≥ 60% Sinus rhythm LVOT gradient < 30 mmHg
METAL‐HCM^7^ (2010)	1:1 perhexiline (100 mg daily): placebo	3–6 months' duration (mean, 4.6 ± 1.8 months)	Suppression of carnitine palmitoyl transferase I and II	Age 18–80 years LV wall thickness ≥15 mm Sinus rhythm LVOT gradient <30 mmHg Exertional symptoms VO_2_ max < 75% predicted
INHERIT^8^ (2015)	1:1 losartan (100 mg daily): placebo	12 months	Inhibition of angiotensin II type 1 receptor	Age ≥18 years LV wall thickness ≥15 or ≥13 mm if family history of HCM LVEF ≥ 50% Sinus rhythm Any LVOT gradient
Ho *et al*.^9^ (2015)	1:1 diltiazem (titrated to 360 mg daily or 5 mg/kg/day): placebo	12–42 months (median 25 months)	Intracellular calcium handling	Age ≥5 years old Normal LV wall thickness (≤12 mm in adults or *z*‐score ≤ 3 in children) Pathogenic or likely pathogenic HCM sarcomeric variant
Coats *et al*.^10^ (2019)	1:1 trimetazidine (20 mg 3 times daily): placebo	3 months	Direct inhibition of fatty acid β‐oxidation	Age ≥18 years LVOT gradient <50 mmHg NYHA class ≥2 V̇O_2_ max < 80% predicted
Maron *et al*.^11^ (2018)	1:1 spironolactone (50 mg daily): placebo	12 months	Normalization of collagen formation through aldosterone receptor blockade	Age 18–55 years HCM diagnosis Any LVOT gradient
HALT‐HCM^12^ (2018)	2:1 n‐acetylcysteine (2400 mg daily): placebo	12 months	Attenuation of interstitial fibrosis through reduction in oxidative stress	Age ≥18 years LV wall thickness ≥15 mm Preserved LV systolic function Any LVOT gradient
RESTYLE‐HCM^13^ (2018)	1:1 ranolazine (2000 mg daily): placebo	5 months	Inhibition of the cardiac late sodium current	Age >18 years LV wall thickness ≥15 mm Sinus rhythm LVOT gradient <30 mmHg NYHA II–III VO_2_ max < 75% predicted
MAVERICK‐HCM^14^ (2020)	1:1:1 mavacamten (titrated to plasma level of 200 ng/mL): mavacamten (titrated to plasma level of 500 ng/mL): placebo	16 weeks	Selective allosteric inhibition of cardiac myosin ATPase	Age ≥18 years LV wall thickness ≥15 or ≥13 mm if family history of HCM LVEF ≥ 55% LVOT gradient ≤30 mmHg NYHA II–III NTproBNP >300 pg/mL
EXPLORER‐HCM^15^ (2020)	1:1 mavacamten (starting dose 5 mg daily, titrated according to LVOT gradient and plasma concentration): placebo	30 weeks	Selective allosteric inhibition of cardiac myosin ATPase	Age ≥18 years LV wall thickness ≥15 or ≥13 mm if family history of HCM LVEF ≥ 55% LVOT gradient ≥50 mmHg NYHA II–III
VANISH^16^ (2021)	Active run in followed by 1:1 valsartan (adults: 320 mg daily; children <18 years weighing ≥35 kg: 160 mg daily; children <18 years weighing <35 kg: 80 mg daily): placebo	2 years	Inhibition of TGF‐β activation by inhibiting angiotensin II type 1 receptor	Age 8–45 years LV wall thickness 12–25 mm LVEF ≥ 55% LVOT gradient ≤30 mmHg NYHA I–II Pathogenic or likely pathogenic HCM sarcomeric variant
VALOR‐HCM^17^ (2022)	1:1 mavacamten (starting dose 5 mg daily, titrated according to LVOT gradient and LVEF): placebo	16 weeks	Selective allosteric inhibition of cardiac myosin ATPase	Age ≥18 years LV septal thickness ≥15 or ≥13 mm if family history of HCM LVEF ≥ 60% LVOT gradient ≥50 mmHg at rest or with provocation NYHA III–IV or II and exertional syncope/near syncope Referred for septal reduction therapy and actively considering scheduling the procedure
REDWOOD‐HCM^18^, ^a^ (2023)	Cohort 1: 1:1 aficamten (5–15 mg): placebo Cohort 2: 1:1 aficamten (10–30 mg): placebo	10 weeks	Selective inhibition of cardiac myosin that acts by binding directly to cardiac myosin at a distinct allosteric binding site	Age 18–85 years LV wall thickness ≥15 or ≥13 mm if family history of HCM LVEF ≥ 60% Resting LVOT gradient ≥50 mmHg or resting LVOT gradient ≥30 and <50 mmHg with post‐Valsalva gradient ≥50 mmHg NYHA II–III
RESOLVE‐HCM^19^ (2021)	1:1 perhexiline (starting dose 100 mg daily, titrated according to plasma concentration): placebo	12 months	Suppression of carnitine palmitoyl transferase I and II	Age ≥18 years Interventricular septal thickness ≥15 mm LVEF ≥ 55% Any LVOT gradient NYHA II/III and/or CCS II/III and treatment with B‐blockers and/or non‐dihydropyridine calcium antagonists and/or disopyramide NT‐proBNP >125 pg/mL
EXPLORER‐CN^20^ (2023)	2:1 mavacamten (starting dose 2.5 mg daily followed by three‐step blinded dose titration guided by core laboratory LVEF, Valsalva LVOT gradient, and plasma drug concentration): placebo	30 weeks	Selective allosteric inhibition of cardiac myosin ATPase	Aged ≥18 years old Diagnosed with oHCM LVOT peak gradient ≥50 mmHg LVEF ≥ 55% NYHA II or III
ODYSSEY‐HCM^21^ (ongoing)	1:1 mavacamten: placebo	48 weeks	Selective allosteric inhibition of cardiac myosin ATPase	LV wall thickness ≥15 or ≥13 mm if family history of HCM Resting LVOT peak gradient <30 and <50 mmHg with provocation NYHA II or III
MEMENTO^22^ (ongoing)	1:1 mavacamten: placebo	48 weeks	Selective allosteric inhibition of cardiac myosin ATPase	Age ≥18 years LV wall thickness ≥15 or ≥13 mm if family history of HCM LVEF ≥ 55% LVOT gradient ≥30 and ≥50 mmHg after Valsalva or exercise NYHA II–III
IMPROVE‐HCM^23^ (2024)	1:1 ninerafaxstat (400 mg total daily dose): placebo	12 weeks	Inhibition of 3‐ketoacyl‐CoA thiolase	Age 18–80 years Diagnosed with non‐obstructive HCM Ability to perform treadmill CPET
SEQUOIA‐HCM^24^ (2023)	1:1 aficamten (5–20 mg daily, titrated according to echocardiography assessment): placebo	24 weeks	Selective inhibition of cardiac myosin ATPase	Age 18–85 years LV wall thickness ≥15 or ≥13 mm if family history of HCM LVEF ≥ 60% Resting LVOT gradient ≥30 mmHg and Valsalva gradient ≥50 mmHg NYHA II–III Respiratory exchange ratio ≥1.05 and VO_2_ max < 80% predicted
ACACIA‐HCM^25^ (ongoing)	1:1 aficamten: placebo	36 weeks	Selective inhibition of cardiac myosin ATPase	18–85 years of age Resting LVOT gradient <30 mmHg and post‐Valsalva <50 mmHg LVEF ≥ 60% VO_2_ max ≤ 90% predicted NT‐proBNP ≥ 300 pg/mL or ≥900 pg/mL if atrial fibrillation or atrial flutter are present at screening, NYHA class II or III KCCQ clinical summary score ≥30 and ≤85
TEMPEST^26^ (Ongoing)	1:1 trientine (800 mg total daily dose): placebo	12 months	Chelation of unbound/loosely bound tissue copper II ions	Age 18–75 years LV wall thickness ≥15 mm LVEF ≥ 50% Any LVOT gradient NYHA I–III

CCS, Canadian Cardiovascular Society; CPET, cardiopulmonary exercise test; HCM, hypertrophic cardiomyopathy; KCCQ, Kansas City Cardiomyopathy Questionnaire; LV, left ventricle; LVEF, left ventricular ejection fraction; LVOT, left ventricular outflow tract; NTproBNP, N‐terminal prohormone of brain natriuretic peptide; NYHA, New York Heart Association; oHCM, obstructive hypertrophic cardiomyopathy; TGF‐β, transforming growth factor beta; VO_2_ max, maximum rate of oxygen consumption.

^a^ Only blinded REDWOOD‐HCM cohorts included.

**Figure 1:**
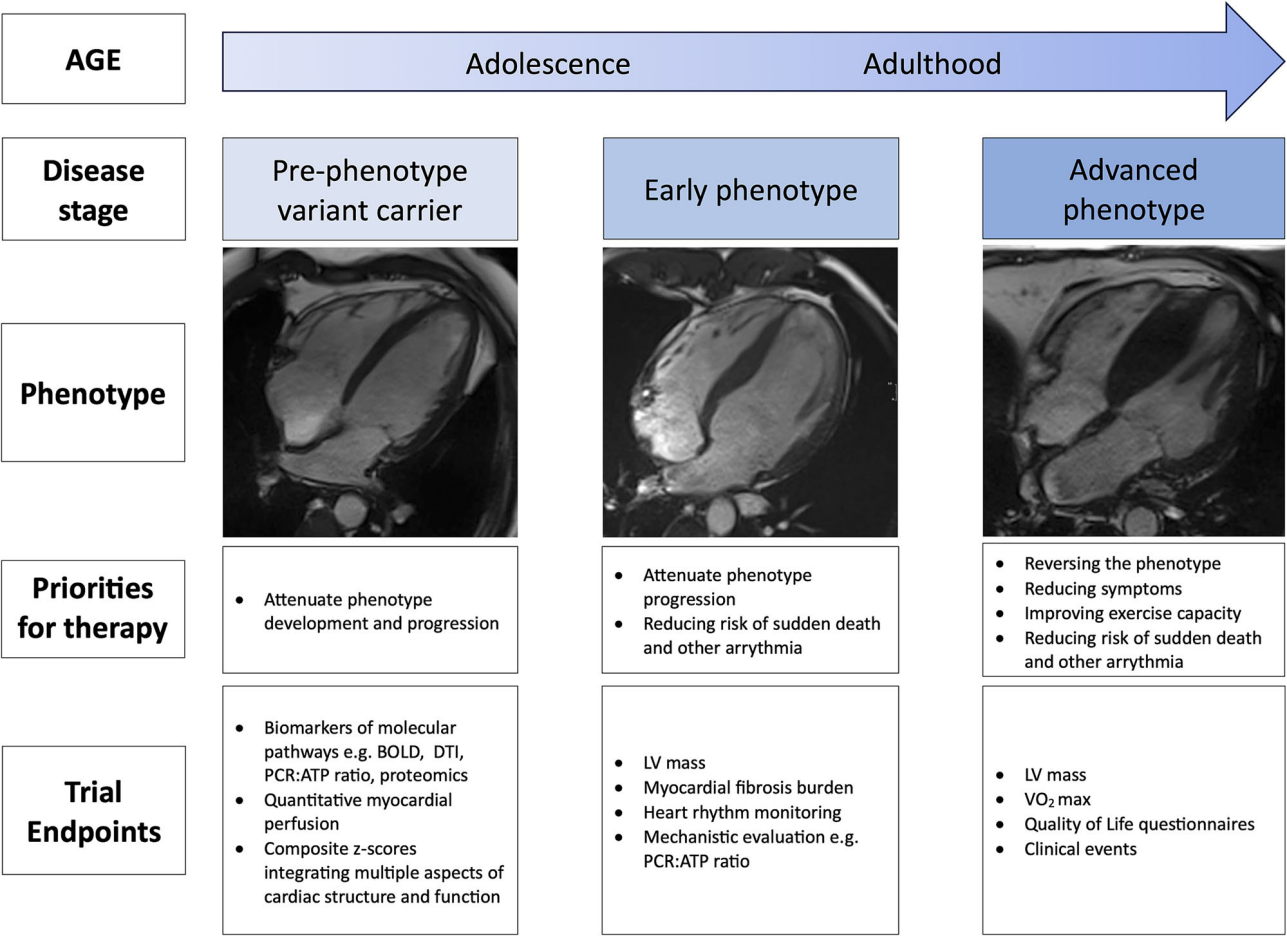
The challenges to consider when designing clinical trials and interpreting findings. BOLD, blood oxygenation level dependent imaging; DTI, diffusion tensor imaging; LV, left ventricle; PCr/ATP, phosphocreatine/adenosine triphosphate; VO_2_ max, maximum rate of oxygen consumption.

HCM poses several unique challenges which are important to recognize when designing clinical trials and interpreting findings (*Figure* *1*). This manuscript discusses the key considerations in the context of recent and ongoing randomized trials.

## Patient selection

The lack of a universally accepted standardized definition of HCM, the heterogenous genotypic and phenotypic nature of HCM and the variety of disease mechanisms or phenotype characteristics being targeted have led to considerable variation in trial enrolment criteria and resultant study populations (*Tables* *1* *and* *2*). Across published trials that have focused on established phenotypic HCM, baseline mean body surface area‐indexed left ventricular mass (LVMi) varies from 106.4 to 142.0 g/m^2^, and mean maximum LV wall thickness (MWT) varies from 16.3 to 23 mm, falling to 49.6 g/m^2^ and 9.0 mm, respectively, for trials that include pre‐hypertrophic genetic variant carriers. Mean peak oxygen consumption (VO_2_ max) varies from 16.4 to 30.0 mL/min/kg, and mean resting LVOT gradient from 7.5 to 89.0 mmHg. In the NHLBI HCM Registry (HCMR), the largest, prospective contemporary HCM cohort, including 2755 patients (44 sites, 6 countries), mean LVMi was 89 ± 27 g/m^2^ in males and 77 ± 25 g/m^2^ in females, mean maximal wall thickness was 18.6 ± 4.8 mm, and 18% of patients had a resting LVOT gradient >30 mmHg, albeit the requirement for cardiac magnetic resonance imaging (CMR) precluded patients with and ICD who typically have more advanced phenotypes.^29^ Importantly, trial populations do not usually represent the variation in age, sex and ethnicity encountered in clinical practice, all of which substantially impact the HCM phenotype, limiting the generalizability of trial findings.^15^

**Table 2:** Baseline characteristics (with weighted means) of patients in completed and ongoing randomized controlled trials in HCM

Trial/authors	Key baseline characteristics		
CHANCE^6^ (2009)		Active (*n* = 12)	Placebo (*n* = 12)
	Age (y)	41 ± 15	45 ± 13
	Male sex (%)	5 (42)	6 (50)
	LV mass, g	407 ± 139	451 ± 228
	Wall thickness, mm	20.0 ± 3.6	20.1 ± 2.5
	VO_2_ max, mL/kg/min	Not available	Not available
	NYHA class, *n* (%)	Class I: 4 (33)	Class I: 4 (36)
		Class II: 4 (33)	Class II: 4 (36)
		Class III: 4 (33)	Class III: 3 (27)
	LVOT gradient, mmHg	7.5 ± 3.1	9.2 ± 6.3
	Genotype positive, *n* (%)	10 (83)	8 (67)
METAL‐HCM^7^ (2010)	Mean ± SE	Active (*n* = 24)	Placebo (*n* = 22)
	Age (y)	56 ± 0.46	54 ± 0.64
	Male sex, *n* (%)	24 (100)	22 (100)
	LV mass, g	Not available	Not available
	Wall thickness, mm	23.2 ± 0.2	22.5 ± 0.1
	VO_2_ max, mL/kg/min	22.2 ± 0.2	23.6 ± 0.3
	NYHA class, *n* (%)	Not available	Not available
	LVOT gradient, mmHg	Not available	Not available
	Genotype positive, *n* (%)	Not available	Not available
	PCr/ATP ratio	1.27 ± 0.02	1.29 ± 0.01
INHERIT^8^ (2015)		Active (*n* = 64)	Placebo (*n* = 69)
	Age (y)	52 ± 12	51 ± 14
	Male sex, *n* (%)	46 (67)	40 (62)
	Indexed LV mass, g/m^2^	108 ± 33	105 ± 42
	Max wall thickness, mm	23 ± 6	23 ± 6
	VO_2_ max mL/kg/min	Not available	Not available
	NYHA class, *n* (%)	Class I: 41 (59)	Class I: 44 (69)
		Class II: 22 (32)	Class II: 18 (28)
		Class III: 6 (9)	Class III: 2 (3)
	LVOT gradient, mmHg (IQR)	21 (10–37)	14 (8–67)
	Genotype positive, *n* (%)	29 (42%)	28 (44%)
Ho *et al*.^9^ (2015)	Mean ± SE	Active (*n* = 19)	Placebo (*n* = 20)
	Age (y)	14.1 ± 1.7	17.3 ± 2.1
	Male sex, *n* (%)	7 (39)	9 (45)
	Indexed LV mass, g/m^2^	49.9 ± 3.8	46.6 ± 3.4
	Max wall thickness mm	8.1 ± 0.4	8.1 ± 0.35
	VO_2_ max, mL/kg/min	Not available	Not available
	NYHA class, n	Not available	Not available
	LVOT gradient, mmHg	Not available	Not available
	Genotype positive, *n* (%)	18 (100)	20 (100)
Coats *et al*.^10^ (2019)		Active (*n* = 27)	Placebo (*n* = 24)
	Age (y)	49 (13)	51 (14)
	Male sex, *n* (%)	18 (67)	18 (75)
	Indexed LV mass, g/m^2^	Not available	Not available
	Max wall thickness, mm	16.5 ± 2.9	16.1 ± 2.9
	VO_2_ max, mL/kg/min	17.4 ± 3.9	17.4 ± 3.6
	NYHA class, *n*	Not available	Not available
	LVOT gradient, mmHg	6.5 ± 4.2	8.2 ± 15.9
	Genotype positive, *n* (%)	Not available	Not available
Maron *et al*.^11^ (2018)		Active (*n* = 26)	Placebo (*n* = 27)
	Age (y)	40 ± 13	42 ± 13
	Male sex, *n* (%)	20 (77)	18 (67)
	Indexed LV mass, g/m^2^	111 ± 26	125 ± 39
	Max wall thickness, mm	22 ± 7	21 ± 6
	VO_2_ max, mL/kg/min	30 ± 7	28 ± 7
	NYHA class, *n* (%)	Class I: 14 (54)	Class I: 13 (48)
		Class II: 9 (35)	Class II: 11 (41)
		Class III: 4 (15)	Class III: 4 (15)
	LVOT gradient, mmHg	11 ± 29	12 ± 28
	Genotype positive, *n* (%)	Not available	Not available
HALT‐HCM^12^ (2018)		Active (*n* = 29)	Placebo (*n* = 13)
	Age	50.7 ± 15.0	47.6 ± 15.1
	Sex (%)	22 (76)	10 (77)
	Indexed LV mass, g/m^2^	128.48 ± 43.60	141.95 ± 50.14
	Wall thickness	20.54 ± 4.42	20.55 ± 3.24
	VO_2_ max, mL/kg/min	Not available	Not available
	NYHA class, *n* (%)	Class I: 16 (55.2)	Class I: 5 (38.5)
		Class II: 11 (37.9)	Class II: 5 (38.5)
		Class III: 2 (6.9)	Class III: 3 (23.0)
	LVOT gradient, mmHg	14.81 ± 25.14	10.45 ± 23.44
	Genotype positive, *n* (%)	26 (68) in all participants; the split between placebo and treatment group was not reported	
RESTYLE‐HCM^13^ (2021)		Active (*n* = 40)	Placebo (*n* = 40)
	Age y	54 ± 14	52 ± 13
	Male sex, *n* (%)	24 (60)	22 (55)
	LV mass, g	Not available	Not available
	Max wall thickness, mm	21.3 ± 6.3	20.3 ± 4.5
	VO_2_ max, mL/kg/min	16.91 ± 5.01	17.23 ± 4.80
	NYHA class, *n*	Not available	Not available
	LVOT gradient, mmHg	9.1 ± 7.1	8.0 ± 5.5
	Genotype positive, *n* (%)	Not available	Not available
MAVERICK‐HCM^14^ (2020)		Pooled active group (*n* = 40)	Placebo (*n* = 19)
	Age (y)	54.0 ± 14.6	53.8 ± 18.2
	Male sex, *n* (%)	19 (47.5)	6 (32)
	LV mass, g	Not available	Not available
	Max wall thickness, mm	18.8 ± 3.5	20.6 ± 4.0
	VO_2_ max, mL/kg/min	20.4 ± 6.0	17.9 ± 5.1
	NYHA class, *n* (%)	Class II: 33 (82.5)	Class II: 13 (68.4)
		Class III: 7 (17.5)	Class III: 6 (31.6)
	LVOT gradient, mmHg	8.8 ± 3.5	7.8 ± 2.5
	Genotype positive, *n* (%)	14 (50.0)	8 (66.7)
EXPLORER‐HCM^15^ (2020)		Active (*n* = 123)	Placebo (*n* = 128)
	Age (y)	58.5 ± 12.2	58.5 ± 11.8
	Male sex, *n* (%)	66 (54%)	83 (65%)
	LV mass, g	Not available	Not available
	Max wall thickness mm	20 ± 4	20 ± 3
	VO_2_ max, mL/kg/min	18.9 ± 4.9	19.9 ± 4.9
	NYHA class, *n* (%)	Class II: 88 (72%)	Class II: 95 (74%)
		Class III: 35 (28%)	Class III: 33 (26%)
	LVOT gradient, mmHg	Rest: 52 ± 29	Rest: 51 ± 32
		Valsalva: 72 ± 32	Valsalva: 74 ± 32
		Post‐exercise: 86 ± 34	Post‐exercise: 84 ± 36
	Genotype positive, *n*/*n* tested	28/90 (31%)	22/100 (22%)
VANISH^16^ (2021)		Active (*n* = 88)	Placebo (*n* = 90)
	Age (y)	23.1 ± 10.1	23.5 ± 10.1
	Male sex, *n* (%)	54 (61)	55 (61)
	Indexed LV mass, g/m^2^	74 ± 23	72 ± 25
	Max wall thickness, mm	17.9 ± 4.7	16.4 ± 3.4
	VO_2_ max mL/kg/min	Not available	Not available
	NYHA class, *n* (%)	Class I: 80 (91%)	Class I: 84 (93%)
		Class II: 8 (9%)	Class II: 6 (7%)
	LVOT gradient, mmHg	Not available	Not available
	Genotype positive, *n* (%)	88 (100)	90 (100)
VALOR‐HCM^17^ (2022)		Active (*n* = 56)	Placebo (*n* = 56)
	Age (y)	59.8 ± 14.2	60.9 ± 10.5
	Male sex, *n* (%)	29 (51.8)	28 (50.0)
	LV mass, g	Not available	Not available
	Max wall thickness, mm	Not available	Not available
	VO_2_ max, mL/kg/min	Not available	Not available
	NYHA class, *n* (%)	Class II with exertional syncope: 4 (7.1)	Class II with exertional syncope: 4 (7.1)
		Class III or higher: 52 (92.9)	Class III or higher: 52 (92.9)
	LVOT gradient, mmHg	Rest: 51.2 ± 31.4	Rest: 46.3 ± 30.5
		Valsalva: 75.3 ± 30.8	Valsalva: 76.2 ± 29.9
	Post‐exercise: 82.5 ± 34.7	Post‐exercise: 85.2 ± 37.0
	Genotype positive, *n* (%)	Not available	Not available
REDWOOD‐HCM^18^ (2023)	Median (IQR)	Pooled active group (*n* = 28)	Pooled placebo (*n* = 13)
	Age y	57 (26–33)	59 (53–64)
	Male sex, *n* (%)	13 (46)	5 (38)
	LV mass, g	Not available	Not available
	Max wall thickness, mm	Not available	Not available
	VO_2_ max, mL/kg/min	Not available	Not available
	NYHA class, *n* (%)	Class II: 17 (61%)	Class II: 11 (85%)
		Class III: 11 (13%)	Class III: 2 (15%)
	LVOT gradient, mmHg	Rest: 53 (42–70)	Rest: 71 (44–94)
		Valsalva: 84 (69–100)	Valsalva: 89 (80–105)
	Genotype positive, *n* (%)	Not available	Not available

	Weighted mean overall^a^	Weighted mean active treatment group	Weighted mean placebo group
Age (y)	47.6	47.7	47.5
Indexed LV mass, g/m^2^	91.8	92.9	90.8
Max wall thickness, mm	19.3	19.5	19.1
VO_2_ max, mL/kg/min	20.1	20.0	20.3
Resting LVOT gradient, mmHg	31.7	31.0	32.5

LV, left ventricle; LVOT, left ventricular outflow tract; NYHA, New York Heart Association; PCr/ATP, phosphocreatine/adenosine triphosphate; SE, standard error; VO_2_ max, maximum rate of oxygen consumption.

^a^ Weight means calculated from available data.

### Genotype

Patients with HCM and a sarcomeric variant have a twofold risk of adverse outcome compared to those without, and including the presence of a pathogenic or likely pathogenic variant in eligibility criteria improves trial specificity.^4^ Indeed, as therapies that target specific molecular pathways emerge, genotyping may become necessary for trial entry. However, only around a third of patients with a contemporary diagnosis of HCM have a sarcomeric variant; thus, requiring a pathogenic or likely pathogenic variant makes trial recruitment more challenging and may impact the generalizability of the findings, particularly for interventions targeting advanced disease.^30^ Recruiting multiple members of the same family, potentially to different arms, is a further complexity to consider.

Reflecting these factors, only two trials have required participants to carry a pathogenic or likely pathogenic sarcomeric variant for entry (Valsartan for Attenuating Disease Evolution in Early Sarcomeric Hypertrophic Cardiomyopathy [VANISH] and Diltiazem Treatment for Pre‐Clinical Hypertrophic Cardiomyopathy Sarcomere Mutation Carriers).^9^, ^16^ These trials were different from most trials in HCM in that they aimed to attenuate disease evolution in early‐stage disease, based on young age and normal LV wall thickness/absence of severe LV hypertrophy or limiting symptoms, hence the requirement for a positive genotype. Recruitment of the 178 randomized participants in VANISH required 17 specialist HCM centres across 4 countries and took 3 years, and recruitment of the 39 randomized participants in the diltiazem trial required three specialist HCM centres and took 4 years.

### Phenotype

The genetic nature of HCM provides a potential opportunity to intervene before the phenotype has developed, thus, intuitively, the potential to prevent the development of clinical manifestations, such as was the hypothesis in VANISH and the Diltiazem trial.^9^, ^16^ However, most trials have targeted more advanced phenotypes because of the associated worse outcomes to increase the likelihood of having a measurable impact on the primary outcome measure and sometimes also because of the nature of the intervention under evaluation.

In keeping with the clinical diagnostic criteria in the 2014 European Society of Cardiology and 2020 American Heart Association/American College of Cardiology Guidelines,^1^, ^2^ most trials have specified a maximal end‐diastolic LV wall thickness of ≥15 mm for entry. Some trials have also included patients with LV wall thickness ≥13 mm if there is a family history of HCM,^14^, ^15^, ^18^ but most have not. VANISH included patients with a wall thickness of ≥12 mm and had an upper limit of 20 mm, although the latter was increased to 25 mm to facilitate enrolment.^31^ CMR imaging is used routinely in many countries to differentiate HCM from its phenocopies.

As per the HCMR, less than 20% of patients with HCM have a resting left ventricular outflow tract (LVOT) gradient ≥30 mmHg,^29^ and most trials have restricted enrolment to patients with a gradient <30 mmHg, although some have not specified (*Table* *1*).^11^, ^12^, ^19^, ^26^ The exceptions are trials evaluating myosin inhibitors, which reduce cardiac contractility by reducing actin‐myosin cross­bridge formation via selectively inhibiting cardiac myosin ATPase. Trials of mavacamten and aficamten have primarily aimed to improve exercise capacity and symptoms by reducing LVOT gradient and have accordingly required participants to typically demonstrate an LVOT gradient ≥50 mmHg at rest or after provocation (Valsalva or exercise), provided resting gradient is ≥30 mmHg.^15^, ^17^, ^18^, ^20^, ^24^, ^32^ No trials have mandated a specific phenotype for entry (i.e. reverse curvature septal hypertrophy, apical, etc.), but trials requiring LVOT obstruction are inevitably enriched with patients with basal septal hypertrophy.

### Symptoms

Similarly, the myosin inhibitor trials have required participants to have exertional symptoms, typically NHYA class II–III, and the VALOR‐HCM trial, which evaluated whether mavacamten allows severely symptomatic patients with obstructive HCM to improve sufficiently such that they no longer meet criteria for, or choose not to undergo, septal reduction therapy, required participants to be in NHYA class III–IV.^17^ Symptom entry requirements vary across other trials (*Table* *1*). In HCMR, a third of patients were in NYHA class II or higher.^29^

## Primary outcome

The primary outcome of phase 3 cardiovascular trials evaluating clinical effectiveness have conventionally comprised composites of clinical events such as death and major non‐fatal episodes such as hospitalization for heart failure, myocardial infarction, stroke, heart transplantation or aborted SCD. Significantly reducing the risk of such events, and thus positively impacting prognosis, has traditionally been the threshold for achieving regulatory approval for new cardiovascular medications in many countries. More recently, however, the 2019 US Food and Drug Agency (FDA) guidance on heart failure endpoints for drug development makes clear that an effect on symptoms or physical function, without a favourable effect on survival or risk of hospitalization, can be a basis for approval.^33^

The low prevalence of HCM, relative to conditions such as heart failure and ischaemic heart disease, and relatively low clinical event rates make it very difficult to conduct event‐driven trials; indeed, no pharmaceutical trials in HCM has used an event‐driven primary outcome. Instead, primary outcome measurements in HCM trials are typically surrogate endpoints, which would generally be considered phase 2 outcome measurements in more prevalent cardiovascular conditions, or symptom/physical function‐based endpoints. There is marked heterogeneity in the choice of primary outcome, with nearly as many different primary outcomes as there are trials (*Table* *3*).

**Table 3:** Primary outcome measures and corresponding minimum change trials were powered to detect

Trial/authors	Primary outcome measure	Minimum
CHANCE^6^ (2009)	Primary outcome not stated	Not reported
METAL‐HCM^7^ (2010)	VO_2_ max	Change in VO_2_ max of 3 mL/kg/min
INHERIT^8^ (2015)	LVMi	Change in LVMi of 12 g/m^2^
Ho *et al*.^9^ (2015)	Global Doppler diastolic (E′) velocity. (changed prior to analysis to ‘a pilot effort to explore a broad range of imaging and biomarker features’)	Not reported
Coats *et al*.^10^ (2019)	VO_2_ max	Change in VO_2_ max of 2 mL/kg/min
Maron *et al*.^11^ (2018)	Serum markers of collagen turnover	Not reported
HALT‐HCM^12^ (2018)	Feasibility assessment including recruitment, retention, compliance, side effects and LV septal thickness	Not reported
RESTYLE‐HCM^13^ (2021)	VO_2_ max	Change in VO_2_ max of 3 mL/kg/min
MAVERICK‐HCM^18^ (2020)	Safety and tolerability of mavacamten	Not reported
EXPLORER‐HCM^15^ (2020)	Composite including VO_2_ max and NYHA class	1.5 mL/kg/min or greater increase in VO_2_ max and at least one NYHA class reduction or a 3.0 mL/kg/min or greater improvement in VO_2_ max and no worsening of NYHA class
VANISH^16^ (2021)	Composite *z*‐score, averaged individual *z*‐scores for change in the following: BSA‐indexed LV mass BSA‐indexed LA volume BSA‐indexed LVEDV BSA‐indexed LVESV BSA‐adjusted maximal LV wall thickness Age‐adjusted tissue Doppler diastolic (E′) velocity Age‐adjusted tissue Doppler systolic (S′) velocity High‐sensitivity troponin T NTproBNP	Standardized effect size of 0.22 (moderate effect) to 0.25 (large effect) for the composite *z*‐score
VALOR‐HCM^17^ (2022)	Composite eligibility for SRT or patient decision to proceed with SRT	50% relative difference between groups
REDWOOD‐HCM^14^ (2023)	Safety and tolerability	Not reported
RESOLVE‐HCM^18^ (2021)	Interventricular septal thickness	Change in interventricular septal thickness of 0.9 mm/year
EXPLORER‐CN^20^ (2023)	Valsalva LVOT gradient	Reduction in LVOT gradient of 30 mmHg
ODYSSEY‐HCM^21^ (Ongoing)	Composite including VO_2_ max and NYHA class	Not reported
MEMENTO^22^ (Ongoing)	Composite including LVMi and left atrial volume index	Participants achieving both a decrease of at least 5 mL/m^2^ in LAVi and a decrease of at least 5 g/m^2^ in LVMI
IMPROVE‐HCM^23^ (2024)	Safety and tolerability	Not reported
SEQUOIA‐HCM^24^ (2023)	VO_2_ max	1.5 mL/kg/min increase in VO_2_ max
ACACIA‐HCM^25^ (Ongoing)	KCCQ clinical summary score	Not reported
TEMPEST^26^ (Ongoing)	LVMi	2.5 g/m^2^ between group change in LVMi

BSA, body surface area; KCCQ, Kansas City Cardiomyopathy Questionnaire; LA, left atrium; LAVi, indexed left atrial volume; LV, left ventricle; LVEDV, left ventricular end‐diastolic volume; LVESV, left ventricular end‐systolic volume; LVMi, indexed left ventricular mass; LVOT, left ventricular outflow tract; NTproBNP, N‐terminal prohormone of brain natriuretic peptide; NYHA, New York Heart Association; SRT, septal reduction therapy; VO_2_ max, maximum rate of oxygen consumption.

## Exercise capacity

VO_2_ max is the most commonly used primary outcome in HCM trials (primary outcome/part of the primary outcome in four randomized controlled trials (RCT)^7^, ^13^, ^15^, ^24^), including the mavacamten for treatment of symptomatic obstructive hypertrophic cardiomyopathy (EXPLORER‐HCM) trial and the Safety, Efficacy, and Quantitative Understanding of Obstruction Impact of Aficamten in HCM (SEQUOIA‐HCM) trial. Specifically, EXPLORER‐HCM used a composite primary outcome measurement of 1.5 mL/kg/min or greater increase in VO_2_ max and at least one NYHA class reduction or a 3.0 mL/kg/min or greater improvement in VO_2_ max and no worsening of NYHA class. The primary outcome measurement SEQUOIA‐HCM comprised VO_2_ max only, powered to a between group difference of 1.5 mL/kg/min.

Exercise capacity parameters, measured during cardiopulmonary exercise testing (CPET), are variably associated with clinical events in HCM, predominantly heart failure‐related events. In a retrospective single‐centre analysis of 1898 consecutive patients with HCM regardless of LVOT gradient (31% had a resting LVOT gradient ≥30 mmHg), VO_2_ max was independently predictive of death due to heart failure or transplantation (hazard ratio [HR] 0.81, 95% confidence interval [CI] 0.77–0.86), although not SCD or ICD therapies.^34^ Minute ventilation to carbon dioxide production (V_E_/VCO_2_), a submaximal exercise parameter that reflects ventilatory response to carbon dioxide production and that is less dependent on physical conditioning and motivation than VO_2_ max, was also independently predictive of the same outcomes. In a retrospective single‐centre analysis of 1005 consecutive patients with predominantly obstructive HCM (85% had a resting LVOT gradient ≥30 mmHg, and 51% underwent surgical myectomy during follow‐up), achieved percentage of age‐ and gender‐predicted VO_2_ max was independently predictive of a composite outcome of death, appropriate ICD therapies, aborted SCD, stroke and heart failure admission, albeit quite weakly (HR 0.96 [0.93–0.98]).^35^ In a prospective multicentre analysis of 620 consecutive patients with HCM (32% had a resting LVOT gradient ≥30 mmHg), VO_2_ max was not independently predictive of a composite endpoint of heart failure death, cardiac transplantation, NYHA III–IV class progression, severe functional deterioration leading to hospitalization for septal reduction and hospitalization for worsening heart failure, although V_E_VCO_2_ was.^36^ In a single‐centre study of consecutive minimally symptomatic patients with obstructive HCM, achieved percentage of predicted VO_2_ max was independently predictive of a composite outcome of death or severe symptoms (NYHA class III or greater, or Canadian Cardiac Society angina class III or greater), albeit again weakly (relative risk 0.98 [0.96–0.99]).^37^

Beyond the possible prognostic information provided by CPET, measures of peak exercise capacity may be a determinant of quality of life (QoL), although data investigating the relationship between exercise capacity and QoL in HCM is limited. In a prospective single‐centre study of 24 patients with HCM, percentage of predicted VO_2_ max achieved showed a modest correlation with Kansas City Cardiomyopathy Questionnaire (KCCQ) overall summary score (*r* = 0.44, *P* = 0.030).^32^

The minimal clinically important difference in VO_2_ max in HCM remains unclear. A change in VO_2_ max of 3 mL/kg/min is commonly used as a ‘clinically relevant’ difference, based on the improvement in VO_2_ max observed (16.2–19.3 mL/kg/min; *P* < 0·05) in 19 patients undergoing septal ablation.^38^ However, in the previously described study by Coats *et al*., a 1 mL/kg/min change in unadjusted VO_2_ max was associated with a HR for death or transplant of 0.79 (0.74–0.83; *P* < 0.001). Conversely, in a recent meta‐analysis by Bayonas‐Ruiz *et al*., patients experiencing an adverse outcome (composite of events such as SCD, heart failure death, all other related mortality, and ventricular arrhythmias) had a VO_2_ max 6.20 (−9.95 to −4.46) mL/kg/min lower than patients with comparable age, LVOT obstruction and degree of hypertrophy who did not experience an event.^39^

Exercise limitation in HCM is multifactorial, including LVOT obstruction, microvascular dysfunction, systolic, diastolic dysfunction and background physical fitness; thus, whilst exercise capacity is clearly a useful outcome measurement, it does not provide insight into whether an intervention has modulated the disease mechanism it was designed to target.

## LV hypertrophy

LV hypertrophy is the defining feature of HCM, and assessments of LV hypertrophy form the primary outcome/part of the primary outcome in at least five previous or ongoing trials.^8^, ^16^, ^19^, ^22^, ^26^ LVMi is consistently associated with adverse outcomes. In a retrospective single‐centre analysis of 187 consecutive patients with HCM (LVOT gradient not stated), LVMi was independently predictive of a composite of all‐cause mortality, heart transplantation, malignant ventricular arrhythmia or appropriate ICD therapy, and a more specific ‘arrhythmia endpoint’, comprising malignant ventricular arrhythmia and appropriate ICD therapy.^40^ There are no good data describing the minimum clinically important difference in LVMi.

Owing to the limited accuracy and reproducibility of LVMi measurement with echocardiography, MWT is commonly used as a surrogate, albeit it is a relatively poor surrogate in HCM (*r* ^2^ 0.38).^41^ Elliot *et al*. showed that increasing wall thickness is associated with higher risk of sudden cardiac death or ICD discharge (Cox regression *P* = 0·029; relative risk per 5 mm increment 1.31 [95% CI 1.03–1.66]).^42^ An earlier study by Spirito *et al*. showed that wall thickness is independently predictive of SCD (relative risk 1.76 [95% CI 1.19–2.60]). A binary MWT of >30 mm conveyed a risk of SCD of 18.2 per 1000 person‐years (95% CI 7.3–37.6).^43^ Oliviotto *et al*. found binary sex‐specific LVMi thresholds (>91 g/m^2^ in males; >69 g/m^2^ in females) to have higher sensitivity for predicting HCM‐related death than binary MWT > 30 mm (sensitivity 100% vs. 41%) but lower specificity (specificity 39% vs. 90%).^41^ MWT is associated with high measurement variability, even with expert analysis of CMR images; AI‐driven automated measurement of LVMi and MWT appears to offer considerably higher precision, thus potentially enabling smaller trial sample sizes, and will likely become standard.^44^, ^45^, ^46^ A recognition for the variation in MWT according to sex and ethnicity is also important.^47^

## Health status

There is increasing focus on developing therapies that improve how patients ‘feel and function’.^33^ The US FDA ‘has qualified Kansas City Cardiomyopathy Questionnaire (KCCQ) as a clinical outcome assessment’ in heart failure.^48^ In patients with HCM, KCCQ overall summary and clinical summary scores show significant, albeit modest correlations with VO_2_ max (*r* = 0.31–0.36) and exercise duration (*r* = 0.35–0.39) and with other questionnaires of breathlessness, tiredness and symptoms (*r* = 0.53–0.68).^48^ In a health status analysis of the EXPLORER trial, 30 weeks of mavacamten was associated with a 9.1‐point improvement in both the overall summary (9.1 [95% CI 5.5–12.8]) and clinical summary scores (9.1 [95% CI 5.5–12.7]) compared to placebo. Thirty‐six per cent of patients receiving mavacamten had a greater than 20‐point improvement in overall and clinical summary scores compared to 15% and 13%, respectively, in the placebo cohort. Following cessation of therapy, mean overall and clinical summary scores returned to baseline at 8 weeks.^49^ The EuroQol Five Dimension (EQ‐5D), a generic assessment of health related QoL, is often preferred by healthcare commissioning bodies because responses have been mapped to healthcare utilities, enabling calculation of quality‐adjusted life years and health economic analysis.^50^ In Explorer HCM, mavacamten was associated with a significant improvement in EQ‐5D‐5L index score compared to placebo (mavacamten = 0.084; placebo = 0.009; adjusted difference = 0.073 [95% CI = 0.027–0.118]).^51^

NYHA class is a physician‐derived metric rather than a patient reported outcome but is included in the FDA endpoint guidance^33^ and formed part of the primary outcome in the EXPLORER trial of mavacamten, where 65% of patients receiving mavacamten experienced a ≥1 NHYA class improvement compared to 31% with placebo.^15^ NYHA class shows a moderate correlation with KCCQ scores in HCM, including overall summary score (*r* = −0.623, *P* = 0.001).^32^

## Mechanistic outcomes

### LVOT gradient

Obstructive HCM (oHCM) is typically defined as a resting or provoked peak LV outflow tract (LVOT) gradient >30 mmHg.^52^ As described, less than one in five patients have oHCM at rest.^29^ Whilst a further third to a half of patients are reported to have inducible LVOT obstruction, studies investigating this have generally been small, highly selective, affected by referral bias and not reflective of contemporary clinical populations.^3^, ^53^ For example, in comparison to the HCMR (18% oHCM at rest), Maron *et al*. reported 41% of patients had oHCM at rest, although HCMR is subject to its own referral bias, as described.^3^ Real‐world prevalence of inducible LVOT obstruction remains unclear. Resting LVOT gradient is independently predictive of all‐cause mortality (HR: 1.005; 95% CI: 1.001–1.009; *P* < 0.01), and oHCM is associated with a higher risk of sudden cardiac death or ICD discharge compared to non‐oHCM (95.7% [95% CI: 93.8–97.6] vs. 91.4% [95% CI: 87.4–95.3]; *P* = 0.0004).^54^, ^55^ Trials of myosin inhibitors, which target LVOT gradient as a key mechanism of action, have used LVOT gradient as a primary outcome at phase 2 and a secondary outcome at phase 3.^22^, ^56^

### Myocardial fibrosis

CMR late gadolinium enhancement (LGE), a marker of focal myocardial fibrosis, is present in approximately half of patients with HCM and associated with adverse outcome.^29^ In a multicentre study of 1293 HCM patients with median 3.3 years follow‐up, extent of LGE was associated with an increased risk of SCD (adjusted HR, 1.46 per 10% increase in LGE; *P* = 0.002), death from any cause and development of end‐stage HCM.^57^ A meta‐analysis by Weng *et al*. evaluating 2993 patients from seven studies showed the binary presence of LGE to be independently predictive of SCD (OR: 3.41; 95% CI: 1.97–5.94; *P* < 0.001), all‐cause mortality (OR: 1.80, 95% CI: 1.21–2.69; *P* = 0.004) and cardiovascular mortality (OR: 2.93, 95% CI: 1.53–5.61; *P* = 0.001).^58^ LGE detection of non‐ischaemic fibrosis requires spatial heterogeneity and is ‘not designed for quantifying fibrosis in non‐infarcted myocardium and is not validated as a quantitative metric for this purpose’.^59^ Its role as a trial outcome measure is therefore uncertain. In a CMR dedicated sub‐study of EXPLORER HCM, mavacamten had no impact on LGE despite improvements in other mechanistic endpoints.^60^

In contrast, the CMR extracellular volume (ECV) technique provides accurate and robust measurement of myocardial fibrosis and has been used as an endpoint in trials of antifibrotic therapies in heart failure where myocardial fibrosis regression has been demonstrated.^61^, ^62^, ^63^ ECV also allows quantification of absolute myocardial extracellular and cellular mass, which may provide more useful assessment of fibrosis regression than ECV in trials of interventions expected to lead to both cardiomyocyte shrinkage and fibrosis regression.^64^ ECV is elevated in HCM and was independently predictive of a composite outcome of cardiovascular death, transplant, aborted SCD and syncope, resulting in cardiopulmonary resuscitation in 263 patients with HCM (HR 1.374 (1.203 to 1.570) per 3% increase in ECV; *P* < 0.001).^29^, ^65^ ECV, absolute myocardial extracellular and cellular mass are secondary outcomes in the phase 2 evaluation of the efficacy and mechanism of trientine in patients with hypertrophic cardiomyopathy (TEMPEST) trial.^26^

### Magnetic resonance spectroscopy

Energy depletion is widely hypothesized to be an integral HCM disease mechanism, via which genetic variants lead to the phenotype.^66^ Impaired myocardial energetics, measured using ^31^phosphorus magnetic resonance spectroscopy to obtain phosphocreatine (PCr) to adenosine triphosphate (ATP) ratio, are observed in HCM sarcomeric variant carriers before developing LVH, and impaired myocardial energetics are associated with LGE progression.^66^, ^67^ PCr:ATP ratio was used as an exploratory endpoint in METAL‐HCM, where perhexiline was associated with an improvement in PCr:ATP ratio (1.27–1.73; *P* = 0.003), and it is a key mechanistic outcome in TEMPEST, but its use in clinical trials has generally been limited due to its lack of widespread availability, expertise required and variability.^7^, ^26^

### Other mechanistic outcomes

Other imaging methods such as blood oxygenation level dependent imaging, diffusion tensor imaging and quantitative myocardial perfusion may also be helpful to evaluate the mechanistic impact of novel interventions.

## Serum cardiac biomarkers

Serum biomarkers of NT‐proBNP and troponin‐T are associated with adverse outcomes in HCM. As a result, NT‐proBNP and troponin‐T have were included in the composite primary endpoint in the VANISH trial. Furthermore, NT‐proBNP has been utilized as an inclusion criterion in the RESOLVE‐HCM and ACACIA‐HCM trials (see *Tables* *1* and *3*). In an observational cohort study of 847 patients with a median follow up of 3.5 years, NT‐proBNP concentration predicted long‐term survival from the primary endpoint of all‐cause mortality or cardiac transplantation (area under the receiver operating characteristic curve of 0.78 [95% CI 0.73–0.84]) and a serum concentration of ≥135 pmol/L was associated with an annual event rate of 6.1% (95% CI 4.4–7.7).^68^ In a single‐centre study of 183 patients with a median follow up of 4.1 years, in a multivariate analysis, high‐sensitivity troponin‐T was an independent predictor of cardiovascular deaths, unplanned heart failure admissions, sustained ventricular tachycardia, embolic events and progression to NHYA functional class III or IV status (HR: 3.23, *P* = 0.012).^69^ Serum cardiac biomarkers are routinely assessed as key secondary outcome measures in HCM trials, most notably in SEQUOIA‐HCM where 24 weeks of aficamten was associated with a geometric mean proportional change of 0.20 (95% CI 0.17–0.22) in the aficamten group and 1.00 (95% CI 0.91–1.07) in the placebo group.^70^

## Composite outcome measurements

Composite outcomes potentially enable smaller sample sizes, which is particularly relevant in HCM.^8^, ^13^, ^16^ The primary outcome of EXPLORER included VO_2_ max and NYHA class, which allowed evaluation of symptom burden and functional capacity, whilst also maximizing the opportunity of detecting a treatment effect and minimizing sample size.^15^ VANISH used a primary outcome that integrated a range of cardiac structural and functional measurements into a composite *z*‐score (*Table* *3*) in view of the study targeting patients with an early phenotype, factors driving disease progression in HCM remaining unclear and the mechanism of action of the intervention (valsartan) in HCM also being unclear. Whilst maximizing the opportunity of detecting a treatment effect, the relative complexity of this approach is likely to make it more difficult to translate the findings into clinical practice.

### Treatment duration

Unlike most phase 3 cardiovascular trials, which are event driven, treatment duration in HCM trials is largely determined by the anticipated time for the intervention to impact the phenotypic trait being targeted (*Table* *1*). Cardiac myosin inhibitors lead to a significant reduction in LVEF by 4 weeks,^56^ enabling the duration of phase 2 trials to be relatively short (10–16 weeks),^14^, ^18^, ^56^ and the duration of larger trials, measuring the impact on exercise capacity and clinical decision making, to also be short (16–30 weeks).^15^, ^17^ In contrast, VANISH had a treatment duration of 2 years.^16^ Treatment duration in TEMPEST (12 months) is informed in part by pilot trial data in diabetes showing the reduction in LVMi with trientine doubled from 6 to 12 months.^71^ Many such trials have longer‐term open‐label follow‐up.^72^

### Example sample size calculations

Notwithstanding the lack of well‐evidenced minimal clinically important differences, exemplar sample size calculations are provided for the most commonly used primary outcome measurements.

#### VO_2_ max

In the EXPLORER trial, mean change in VO_2_ max from baseline was 1.4 ± 3.1 mL/kg/min in the mavacamten group and −0.1 ± 3.0 mL/kg/min in the placebo group (mean ± standard deviation).^15^ Using these data, 66 patients per group provide 80% power to detect a minimum difference in change in VO_2_ max from baseline to follow‐up between active and placebo groups of 1.5 mL/kg/min (two‐sided alpha 0.05), that is, total sample size of 132 patients. To allow for treatment discontinuation in 10%, this could be inflated to 74 patients per group (i.e. total study *n* = 148).

#### LVMI

In a pilot study of trientine in HCM, standard deviation of within‐patient differences in LVMi from baseline to follow‐up was 4.5 g/m^2^ in the trientine group and 2.4 g/m^2^ in the observational control group.^73^ Using a standard deviation of within‐patient differences from baseline of 5 g/m^2^ in both groups, 64 patients per group provide 80% power to detect a minimum difference in change in LVMI from baseline to follow‐up between active and placebo groups of 2.5 g/m^2^ (two‐sided alpha 0.05), that is, total sample size of 128 patients. To allow for treatment discontinuation in 10%, this could be inflated to 72 patients per group (i.e. total study *n* = 144).^26^

### Illustrative sample size for clinical events

To achieve 90% power at 5% significance, 844 first events are required to detect a clinically relevant HR of 0.80. Assuming a 5.6% annual first event rate and a mean follow‐up of 3.5 years, 5224 patients would need to be randomized between the intervention and control. Allowing for 15% loss to follow‐up, 6200 patients are required (3100 per group). With an estimated randomization rate of 0.7 patients per site per month (estimated from HCMR recruitment data), 150 sites would be required. The data behind this power calculation are presented in the Supporting Information.

## Discussion

Trials in HCM are heterogenous, both in the range of putative disease mechanisms targeted and in their design, with varied entry criteria, outcome measures, effect sizes and treatment duration.

This heterogeneity reflects the nature of HCM itself, which encompasses a broad and diverse clinical spectrum, and the lack of detailed understanding of causal mechanisms. Whereas HCM was traditionally considered a monogenic disorder, it has become clear that those carrying sarcomeric variants are the minority.^29^ Recent genome‐wide association analyses have demonstrated the polygenic nature of HCM, particularly sarcomere‐negative HCM, and the causal role of acquired conditions such as diastolic hypertension.^74^ Nevertheless, the underpinning molecular pathways remain unclear, and the phenotypic heterogeneity unexplained.

The limited understanding of HCM pathogenesis has stymied systematic investigation of biological targets for therapeutic intervention. Trialled interventions have typically aimed to modulate more generic, macroscopic disease mechanisms, such as energy deficiency, myocardial fibrosis and calcium handling.^7^, ^9^, ^12^ Whilst it is encouraging that there may be a range of disease manifestations to target, it is unsurprising that until recently, no therapy has proven to be an efficacious modifier of the disease.

The low clinical event rates have precluded event‐driven trials. Trials have therefore conventionally used surrogate outcome measures, such as exercise capacity and LV mass. However, there remains a paucity of evidence for these measurements being independently predictive of clinical events, minimum clinically important differences are poorly defined, and they are often multi‐determined, commonly influenced by factors other than the putative targeted mechanism.^75^ The relationship between different phenotypic features is also poorly characterized. This is particularly relevant for non‐obstructive HCM where choice of primary outcome is challenging, especially in light of the endpoints that the FDA considers acceptable^33^; for example, could a reduction in LV mass be expected to translate into improved exercise capacity or QoL? The lack of understanding of the biological pathways involved has also precluded identification of potential novel endpoints that could accelerate more focused trials. The inclusion of patient‐reported outcome measures in the FDA trial endpoint guidance is an important step forward for patients with symptomatic disease.

In many ways, the myosin inhibitor programme is an exemplar for drug development. Recognizing the fundamental role of excessive myocardial contractility, Green *et al*. identified mavacamten from a chemical screen for molecules that reduce sarcomere contractile function.^76^ Target efficacy was straightforwardly measurable using LVOT gradient, and the relationship between LVOT gradient and trial outcome measures that are important for patients and drug licensing (e.g. exercise capacity and QoL) is also relatively straightforward.^56^ These outcome measures enabled a comparatively small phase 3 sample size, and the rapid onset of action allowed trial duration to be short.^15^ Factors such as these facilitated the remarkable pace of the programme, taking only 6 years from preclinical evaluation to licensing. The long‐term impact of myosin inhibitors on myocardial structure and function is under investigation.^22^

There is, nevertheless, an urgent need for other disease‐modifying therapies, particularly for non‐obstructive HCM, which comprises the majority of the population, and for preventing phenotype expression and progression in early disease. Fundamental to developing new therapies is the need for a better understanding of HCM. This requires a co‐ordinated approach, with large, prospective studies collecting comprehensive multimodal phenotypic and genotypic data linked to health‐related outcomes. Studies such as that by Trados *et al*., which highlighted that a subset of genes underlies both monogenic and polygenic forms of HCM and found evidence for the role of downstream remodelling pathways, demonstrate the value of a co‐ordinated approach, and HCMR in particular.^77^ Genome editing techniques to correct pathogenic variants show promise in preclinical studies for preventing development of the phenotyping although such ‘once in a lifetime’ treatments will require specific trial design.^78^, ^79^ Biomarkers of molecular pathways are required to target interventions appropriately. Important too is the need for comprehensive evaluation as standard in clinical trials in HCM (including CMR, CPET and QoL), which the relatively small sample sizes should allow. Such assessments are currently being conducted as part of the TEMPEST trial.^26^

Non‐obstructive HCM is a specific cause of heart failure in the context of a preserved left ventricular ejection fraction, and it may be trials in HCM and heart failure with preserved ejection fraction (HFpEF) can inform one another. Indeed, trials of myosin inhibitors in HFpEF and trials of sodium‐glucose transport protein 2 inhibitors in HCM are ongoing.

In conclusion, HCM poses a number of challenges for clinical trials. Fundamental to the development and evaluation of novel therapeutics is an improved understanding of HCM itself. Nevertheless, there remains a substantial unmet need, and the success of the myosin inhibitor programme serves to demonstrate that drug development for HCM is highly attractive for investment.

## Funding

WGN is funded by the NIHR Manchester Biomedical Research Centre (NIHR203308). AS is funded by a National Institute for Health and Care Research (NIHR) Advanced Fellowship (NIHR300867). BR is funded by Wellcome Career Development Award fellowship (302210/Z/23/Z) and acknowledges support from the NIHR Oxford Biomedical Research Centre and BHF Centre of Research Excellence, Oxford. HW acknowledges support from the BHF Centre of Research Excellence and from CureHeart and the British Heart Foundation Big Beat Challenge award (BBC/F/21/220106). CAM (Advanced Fellowship, NIHR301338) is funded by the NIHR. The views expressed in this publication are those of the authors and not necessarily those of the NIHR, NHS or the UK Department of Health and Social Care. CAM acknowledges support from the University of Manchester British Heart Foundation Accelerator Award (AA/18/4/34221) and the NIHR Manchester Biomedical Research Centre (NIHR203308).

## Disclosures

WGN is a co‐founder of Fava Health Ltd. HW reports receiving consultancy fees from Cytokinetics, BioMarin and BridgeBio. CAM has participated on advisory boards/consulted for AstraZeneca, Boehringer Ingelheim and Lilly Alliance, Novartis and PureTech Health; serves as an advisor for HAYA Therapeutics; and has received speaker fees from AstraZeneca, Boehringer Ingelheim and Novo Nordisk, conference attendance support from AstraZeneca and research support from Amicus Therapeutics, AstraZeneca, Guerbet Laboratories Limited, Roche and Univar Solutions B.V.

## Supporting information


**Table S1.** Annualised clinical event rates in HCM.
**Table S2.** Distilled composite outcome, component event rates and overall event rate.
**Table S3.** Example sample size calculation parameters.
**Table S4.** Estimated trial recruitment rate based on HCMR Registry data.
**Table S5.** Illustrative example of number of sites and trial duration required.
