# Supplementary material for: Considerations for drug trials in hypertrophic cardiomyopathy
Source: ESC Heart Fail. 2024 Oct 27;12(2):1095–112. doi: 10.1002/ehf2.15138 (PMC11911595; doi:10.1002/ehf2.15138)
Supplement: Supplementary file 1 — Table S1. Annualised clinical event rates in HCM. Table S2. Distilled composite outcome, component event rates and overall event rate. Table S3. Example sample size calculation parameters. Table S4. Estimated trial recruitment rate based on HCMR Registry data. Table S5. Illustrative example of number of sites and trial duration required. [file EHF2-12-1095-s001.docx]

**Considerations for clinical trials in hypertrophic cardiomyopathy**

**Supplementary Material**

**Data to inform the illustrative clinical event-driven sample size calculation**

**Tables**

1. Annualised clinical event rates in HCM
2. Distilled composite outcome, component event rates and overall event rate
3. Sample size calculation
4. Estimated recruitment rate based on data from the HCMR Registry
5. Trial duration calculation

**Table 1. Annualised clinical event rates in HCM**

| **Paper** | Maron 2015^(1)^ | Maron 2015^(2)^ | Coats 2015^(3)^ | Maron 2016^(4)^ | Rowin 2017^(5)^ | Choi 2022^(6)^ | | O'Mahony 2014^(7)^ | Kim 2021^(8)^ | Choi 2022^(9)^ | Chan 2014^(10)^ | Ciabatti 2020^(11)^ | **Mean** | **Weighted mean** |
| --- | --- | --- | --- | --- | --- | --- | --- | --- | --- | --- | --- | --- | --- | --- |
| Cardiovascular death | 0.53 |  | 1.1 | 0.53 |  | | 0.85 |  | 0.54 |  |  |  | 0.71 | 0.74 |
| Stroke death | 0.03 |  |  |  |  | |  |  |  |  |  |  | 0.03 | 0.03 |
| Heart failure death | 0.24 |  | 0.5 | 0.25 |  | |  |  |  |  | 0.14 |  | 0.28 | 0.32 |
| Sudden cardiac death | 0.24 | 0.62 | 0.38 | 0.2 |  | |  | 0.56 |  |  | 0.33 |  | 0.39 | 0.45 |
| Post-surgical death |  |  |  | 0.06 |  | |  |  |  |  |  |  | 0.06 | 0.06 |
| Thromboembolic death |  |  |  |  | 0.1 | |  |  |  |  |  |  | 0.10 | 0.10 |
| Combined stroke, MI, VTE and Post-surgical death |  |  | 0.23 |  |  | |  |  |  |  |  |  | 0.23 | 0.23 |
| Aborted SCD | 0.5 | 0.76 | 0.32 |  |  | |  | 0.38 |  |  | 0.54 |  | 0.50 | 0.47 |
| Transplant | 0.25 |  | 0.21 | 0.19 |  | |  |  |  |  | 0.21 |  | 0.22 | 0.22 |
| All-cause hospitalisation |  |  |  |  |  | | 8.2 |  |  |  |  |  | 8.20 | 8.20 |
| Acute cardiovascular hospitalisation |  |  |  |  |  | |  |  |  |  |  | 1.96 | 1.96 | 1.96 |
| Heart failure hospitalisation |  |  |  |  |  | |  |  | 1.92 |  |  | 0.31 | 1.12 | 1.88 |
| Ischaemia hospitalisation |  |  |  |  |  | |  |  |  |  |  | 0.33 | 0.33 | 0.33 |
| Stroke hospitalisation |  |  |  |  |  | |  |  |  |  |  | 0.23 | 0.23 | 0.23 |
| Atrial Fibrillation hospitalisation |  |  |  |  |  | |  |  |  |  |  | 0.92 | 0.92 | 0.92 |
| Septal reduction therapy |  |  | 2.1 | 1.77 |  | |  |  |  |  |  |  | 1.94 | 1.92 |
| Progressive heart failure symptoms |  |  |  | 3.3 |  | |  |  |  |  | 2.32 |  | 2.81 | 2.90 |
| New AF |  |  |  | 2.97 | 4.1 | |  |  |  | 0.46 |  |  | 2.51 | 1.35 |
| Stroke |  |  |  | 0.12 |  | |  |  |  | 0.65 |  | | 0.39 | 0.55 |
| VTE |  |  |  | 0.12 |  | |  |  |  |  |  |  | 0.12 | 0.12 |

Note: Some values are estimated from the available published data and may not be accurate. Some patients contribute to multiple papers from the same research groups.

**Table 2. Distilled composite outcome, component event rates and overall event rate**

| **Outcomes** | **Annualised event rate** |
| --- | --- |
| CV death | 0.74 |
| Aborted SCD | 0.47 |
| Transplant | 0.22 |
| CV hospitalisation | 1.96 |
| Progressive HF symptoms (I/II to III/IV) | 2.90 |
| New AF | 1.35 |
| **Total** | **7.64** |
| Estimated competing event rate* | 2.00 |
| **Overall estimated annualised event rate** | **5.64** |

*Some outcomes will compete (e.g. progressive HF symptoms and CV hospitalisation, new AF and CV hospitalisation, new AF and progressive HF symptoms), therefore an estimated competing event rate of 2% is deducted from the rate summed from the individual components to provide an overall estimated annualised event rate.

**Table 3. Example sample size calculation parameters**

| Power (%) | 0.9 |
| --- | --- |
| Significance (%) | 0.05 |
| Hazard ratio | 0.8 |
| Required events | 844 |
| Estimated yearly event rate (%) | 5.6 |
| Mean follow up time (years) | 3.5 |
| Required patient number | 5224 |
| Estimated lost to follow up (%) | 0.15 |
| **Total required patient number** | **6200** |

**Table 4 Estimated trial recruitment rate based on HCMR Registry data**

|  |  |
| --- | --- |
| Number of recruited HCMR patients | 2755 |
| Number of sites | 44 |
| Mean recruited per site | 62.61 |
| Duration of recruitment (months) | 36 |
| Mean recruitment rate per site per month | 1.74 |
| **Projected RCT recruitment rate*** | **0.70** |

*****The projected RCT recruitment rate is based upon the assumption that 40% patients that have agreed to be part of an observational registry will agree to participate in an RCT.

**Table 5 Illustrative example of number of sites and trial duration required**

|  |  |
| --- | --- |
| Number of patients recruited per site per month | 0.70 |
| Number of sites | 150 |
| Total recruitment per month | 105 |
| Sample size | 6200 |
| Recruitment duration (months) | 59.05 |
| Recruitment duration (years) | 4.92 |
| Mean follow up during recruitment (years) | 2.46 |
| Additional required follow up to provide 3.5 years means follow-up (years) | 1.04 |
| **Total study duration (years)** | **5.96** |

**Supplemental References**

1. Maron BJ, Rowin EJ, Casey SA et al. Hypertrophic Cardiomyopathy in Adulthood Associated With Low Cardiovascular Mortality With Contemporary Management Strategies. J Am Coll Cardiol 2015;65:1915-28.

2. Maron BJ, Casey SA, Chan RH, Garberich RF, Rowin EJ, Maron MS. Independent Assessment of the European Society of Cardiology Sudden Death Risk Model for Hypertrophic Cardiomyopathy. Am J Cardiol 2015;116:757-64.

3. Coats CJ, Rantell K, Bartnik A et al. Cardiopulmonary Exercise Testing and Prognosis in Hypertrophic Cardiomyopathy. Circ Heart Fail 2015;8:1022-31.

4. Maron MS, Rowin EJ, Olivotto I et al. Contemporary Natural History and Management of Nonobstructive Hypertrophic Cardiomyopathy. J Am Coll Cardiol 2016;67:1399-1409.

5. Rowin EJ, Hausvater A, Link MS et al. Clinical Profile and Consequences of Atrial Fibrillation in Hypertrophic Cardiomyopathy. Circulation 2017;136:2420-2436.

6. Choi Y-J, Kim B, Lee H-J et al. Emergency department utilization in patients with hypertrophic cardiomyopathy: a nationwide population-based study. Scientific Reports 2022;12:3534.

7. O'Mahony C, Jichi F, Pavlou M et al. A novel clinical risk prediction model for sudden cardiac death in hypertrophic cardiomyopathy (HCM risk-SCD). Eur Heart J 2014;35:2010-20.

8. Kim M, Kim B, Choi Y-J et al. Sex differences in the prognosis of patients with hypertrophic cardiomyopathy. Scientific Reports 2021;11:4854.

9. Choi Y-J, Kim B, Rhee T-M et al. Augmented risk of ischemic stroke in hypertrophic cardiomyopathy patients without documented atrial fibrillation. Scientific Reports 2022;12:15785.

10. Chan RH, Maron BJ, Olivotto I et al. Prognostic Value of Quantitative Contrast-Enhanced Cardiovascular Magnetic Resonance for the Evaluation of Sudden Death Risk in Patients With Hypertrophic Cardiomyopathy. Circulation 2014;130:484-495.

11. Ciabatti M, Fumagalli C, Beltrami M et al. Prevalence, causes and predictors of cardiovascular hospitalization in patients with hypertrophic cardiomyopathy. Int J Cardiol 2020;318:94-100.
